# New Insight of Scaffold Based on Hydroxyapatite (HAp)/Bacteria's Nanocellulose (BN) for Dental Tissue Engineering

**DOI:** 10.1055/s-0043-1776123

**Published:** 2023-11-23

**Authors:** Silvia Anitasari, Hendrik Setia Budi, Yung-Kang Shen, Yuliana Mahdiyah Da'at Arina

**Affiliations:** 1 Department of Dental Material and Devices, Dentistry Program, Faculty of Medicine, Universitas Mulawarman, Samarinda, Indonesia; 2 Department Medical Microbiology, Medical Program, Faculty of Medicine, Universitas Mulawarman, Samarinda, Indonesia; 3 Department of Oral Biology, Dental Pharmacology, Faculty of Dental Medicine, Universitas Airlangga, Surabaya, Indonesia; 4 School of Dental Technology, College of Oral Medicine, Taipei Medical University, Taipei, Taiwan; 5 Department of Periodontics, Faculty of Dentistry, University of Jember, Jember, Indonesia

**Keywords:** scaffold, dental tissue engineering, human well-being, physical properties

## Abstract

**Objective** Bacterial nanocellulose (BN), derived from *Acetobacter xylinum* ATCC 237672, is a polymer that offers several desirable characteristics for scaffolds applications. To further enhance the characteristic of the BN scaffold, hydroxyapatite (HAp) from *Anadara granosa* and *Achatina fulica* can be incorporated. Therefore, the aim of the study was to characterize the physical properties of a three-dimensional (3D) scaffold made of HAp and BN.

**Materials and Methods** The scaffold was developed using the cellulose immersion technique, where BN was soaked in HAp suspension for different duration (5, 10, 15, 20, and 25 hours). The physical properties that were evaluated included porosity, pore density, swelling ratio, and water retention.

**Results** The HAp/BN 3D scaffold, which is considered a hydrogel material, exhibited favorable physical properties that can support cell survival. The total porosity of the scaffolds was 100%. There was no significant difference porosity among the groups ( *p* > 0.05). The swelling ratio increased on day 1 and then sharply decreased on day 2. There was a significant difference between the groups on both day 1 and day 2 ( *p* < 0.05). The scaffolds immersed in the HAp for more than 15 hours exhibited higher water retention compared to the other groups, and there was a significant difference between the groups on day 2 and day 4 ( *p* < 0.05). The scaffold immersed for more than 15 hours exhibited a higher pore density compared to those immersed for less than 15 hours, and there was no a significant difference between the groups ( *p* > 0.05).

**Conclusion** Our findings suggest that the HAp/BN 3D scaffold, especially when immersed in HAp for 15 hours, possesses promising physical properties that make it suitable for various applications in dental tissue engineering.

## Introduction

Dental tissues such as enamel, dentin, pulp, cementum, periodontal ligament, and alveolar bone are indeed susceptible to damage and degradation due to various oral conditions. Tissue engineering has emerged as a promising approach to address these issues, and scaffolds play a crucial role in providing a suitable microenvironment for tissue regeneration. 1 Scaffolds used in dental tissue engineering serve as frameworks that support cell attachment, proliferation, and differentiation, mimicking the natural extracellular matrix. They provide mechanical support, guide tissue growth, and facilitate nutrient and waste exchange. Hydrogels are particularly attractive for this purpose due to their high water content, which is similar to the native tissue environment. They are also compatibility with cellular processes and can be designed to degrade over time as the new tissue forms. 2

However, achieving precise control over hydrogel properties, including porosity, remains a challenge. Porosity is a critical parameter as it affects cell infiltration, nutrient diffusion, and waste removal within the scaffold. Proper porosity is required to support cell growth and tissue regeneration effectively. 2 3 Bacterial nanocellulose (BN) is a type of hydrogel material that is produced from *Acetobacter xylinum* bacteria. 3 *A. xylinum* is a Gram-negative, acetic acid bacterium that has shown promise in various medical applications, such as artificial skin, blood vessels, and bone regeneration. BN stands out due to its higher purity and crystalline nanofibrillar structure, which provides a wide surface area capable of retaining a large volume of liquid. Additionally, BN possesses unique properties, including versatility, *in situ* moldability, biocompatibility, biodegradability, high water-holding capacity, cost-effective processing, and high mechanical strength in the wet state. 4 5

Incorporating BN into hydroxyapatite (HAp) can enhance the biocompatibility of HAp which is highly desirable in the field of biomaterials. The shells of *A. granosa* and *A. fulica* contain a high percentage of calcium carbonate (CaCO _3_ ) (98%), 0.3% of calcium phosphate, and chitin (C _8_ H _13_ O _5_ ). 6 7 Chitin has been reported to possess anticancer and antibacterial effects, making it particularly valuable for promoting biocompatibility. There are difference percentage of chitin in the shells of *A. granosa* and *A. fulica* . The shells of *A.* *granosa* contain 14 to 35% chitin, while *A. fulica* shells have a higher chitin content of 70 to 80%. Moreover, the content of the shells of these species does not appear to be significantly influenced by the region they inhabit. This suggests that environmental factors or habitat variations may not play a major role in determining the chitin content of their shells. 8

Incorporating both *A. granosa* and *A. fulica* shells and coating them on the surface of BN could provide an advantage in terms of improving the characteristic and biocompatibility of the scaffold. 7 However, it is important to note that there is limited information regarding the physical characteristics of this composite material, such as porosity, pore density, and swelling ratio. These properties are crucial factors that influence the biocompatibility of the scaffold. Therefore, the aim of the study was to measure the physical properties of the HAp/BN three-dimensional (3D) scaffold, providing valuable insights into its suitability for various biomaterials applications.

## Materials and Methods

### Collection and Identification of *A. granosa* and *A. fulica*

The sampling and collection of *A. granosa* and *A. fulica* for the study were conducted in Samarinda, East Kalimantan, Indonesia. The identification of collected samples was performed at the Ecology and Animal Systematic Laboratory, Universitas Mulawarman, Samarinda, East Kalimantan, Indonesia.

### Bacteria

The study used the *A. xylinum* ATCC 237672 for the production of BN. The bacteria underwent two rounds of culture. The first round of culture was conducted to grow the bacteria's starter culture, and the second round was performed to produce nanocellulose. 9

### Synthesis of Starter Bacteria

*A. xylinum* ATCC 237672 was cultured using a medium consisting of 2.5 g/L of sucrose (342.3 g/mol), 0.5 g/L ammonium sulfate, and water from *Cocos nucifera* (coconut water). The prepared culture medium, containing these ingredients, was poured into sterile container and then sealed. The container was subsequently stored at room temperature for a period 5 to 7 days. This allowed for the growth and cultivation of *A. xylinum* , facilitating the production of BN. 10

### Synthesis of Bacteria Nanocellulose

BN was synthesized by *A. xylinum* ATCC 237672. Seventy-five grams of sucrose was added to 1 L of coconut water ( *C. nucifera* ). The mixture was poured into a beaker glass and filtered. The filtered solution was then boiled in a closed beaker glass at 100°C. After cooling, 5 mL of distilled acetate acid was added to the solution. The solution was filtered through a plastic tray filter (1 tray/L). A 10% starter culture of *A. xylinum* was applied to the filtered solution. The container containing the solution was covered with aluminum foil. The solution was incubated for 7 to 9 days at room temperature to allow the BN to develop. After the incubation period, the BN was cleaned by rinsing it under running water and immersed in water for 2 to 3 days to remove any odor. The water was replaced daily during this immersion period. On the fourth day, the BN was immersed in a 0.5% NaOH solution for 24 hours. After NaOH treatment, the BN was washed several times with water until the pH reached neutrality (pH 7.2). Finally, the nanocellulose was boiled for an additional 15 minutes. 10

### Calcination of Hydroxyapatite from *A. granosa* and *A. fulica*

The shells of *A. granosa* and *A. fulica* were cleaned and dried. Then, the dried shells underwent calcination twice. The first calcination was conducted at the temperature of 800°C for 12 hours to obtain calcium. The second calcination was carried out at the temperature of 800°C for 24 hours. This calcination process helps convert the calcium carbonate (CaCO _3_ ) present in the shells into calcium oxide (CaO), which is a precursor for HAp synthesis. 9

HAp synthesis was carried as follows: a 1-M Ca (OH) _2_ solution was prepared by weighing 24.81 g of CaO and adding it to a beaker glass containing 600 mL of distilled water. The solution was stirred using a magnetizer at a speed of 150 revolutions per minute (rpm) until homogenous. Once homogenized, 300 mL of 1.8 M phosphoric acid was added to the solution. The solution was heated on a hot plate at 400°C and stirred for 60 minutes at 300 rpm to facilitate the reaction between calcium hydroxide and phosphoric acid. After stirring, the solution was allowed to condense for 24 hours. The product was then filtered using a Whatman membrane no. 11 and dried at 100°C for 3 hours. Finally, the dried product underwent the sintering process which was carried out at 900°C for 5 hours. 10

### Fabrication of Hydroxyapatite

Note that 24.81 g of CaO was added into a beaker gas and mixed with 600 mL of distilled water and then stirred at 150 rpm. The mixture was added to 300 mL of phosphoric acid (1.8 M) and then heated on the hot plate with temperature of 400°C. After cooling, the solution was mixed using a stirrer magnet instrument at a speed of 300 rpm for 60 minutes. The solution was aged overnight (for approximately 24 hours). The aged solution was filtered using a Whatman membrane no.11. The resulting CaO product was dried at 1,000°C for 3 hours.

### Purification of Bacterial Nanocellulose

The BN was immersed in deionized (DI) water for 2 days; the DI water was replaced every 5 hours. This helps in the removal of impurities and contaminants from BN. After the immersion in distilled water, the BN was transferred to a 1-M NaOH solution and was immersed for 2 hours. During this time, salt deposits could be on the surface of BN. The BN, along with the salt deposits, was then rinsed with DI water. The rinsing process continued until the pH of water reached 7.2. Additionally, the rinsing process continued until the odor associated with the BN was completely gone and removed it using DI water until the pH was 7.2 and odor was gone. 10

### Synthesis of HAp/Bacteria Nanocellulose

Synthesis of HAP/BN was performed by *cellulose immersion technique* . One gram of HAp powder was added to 30 mL of DI water and then the mixture was sonicated for 30 minutes to disperse the HAp particles evenly in the water. The purified BN obtained after the purification process was immersed in the HAp suspension. While immersing the BN, stirring was conducted using a magnetizer at a speed of 200 rpm for 5, 10, 15, 20, and 25 hours. The pellicle was putted out and was dried at 500°C in oven for 1 day. 10

### Porosity

The porosity of the scaffold was evaluated using ethyl alcohol (EtOH) displacement. The initial volume of EtOH was V1. The total volume of EtOH after the immersion of the scaffold was V2 _._ The residual EtOH volume after scaffold removal was V3 _._ The porosity was calculated using the equation 11 :



### Swelling Ratio

The swelling ratio was calculated using phosphate-buffered saline (PBS) 1× (Gibco, United States) for 4 days. The scaffolds were soaked in the PBS 1X solution at 37°C for 4 days. At predetermined intervals, the scaffolds were withdrawn from the solution and weighted. The swelling ratio was determined using the equation 12 :





where *W _t_* is the weight of the swollen scaffolds at the time intervals, *W _o_* is the weight of dried scaffolds, and *W _d_* is the initial weight of the swollen scaffolds.

### Pore Density

Density is the mass of a unit volume of material substance. The formula for density is:



where *D* is the density, *M* is the mass of scaffolds, and *V* is the volume of scaffolds. 13

### Morphology and Pore Size

The scaffold morphology and pore sizes were measured using scanning electron microscopy (SEM) at an accelerating voltage of 20 kV (SEM; FEI, United States). The pores in the SEM images were analyzed using Image-J software. Scale bars were placed in the SEM image to measure the sizes of the pores, using a known distance as a reference. The contour of a pore was subsequently delineated and measured in micrometers (μm). 14

### Statistical Analysis

All data in the graphs were presented as mean ± standard error of the mean for each group of samples. One-way analysis of variance (ANOVA) was performed using IBM SPSS statistical software (version 21, IBM Company, Armonk, New York, United States) to determine the statistical difference. After performing the ANOVA, a post hoc Tukey's test was performed to determine the statistical difference between the groups. The significance level was set at *p* < 0.05. 15

## Results

**Fig. 1:**
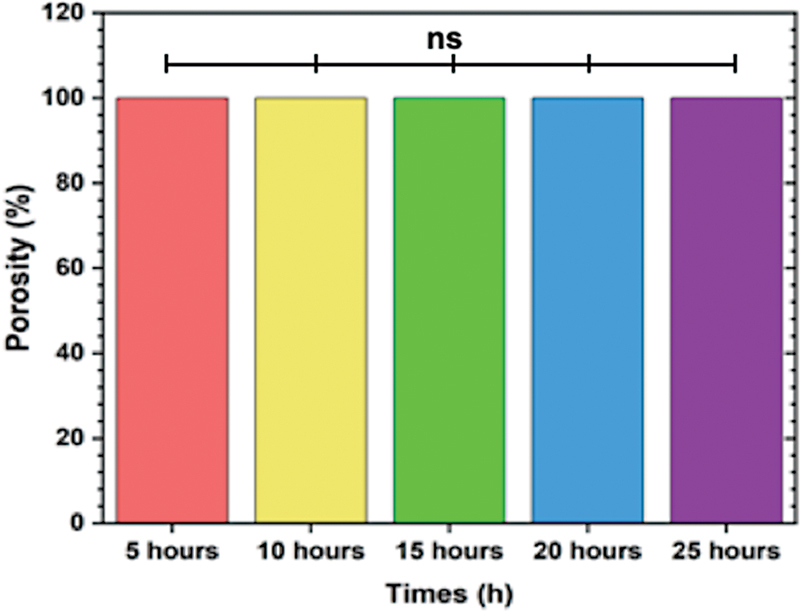
The porosity of hydroxyapatite (HAp)/bacterial nanocellulose (BN) three-dimensional (3D) scaffolds with different time for immersion technique. The result indicated that there was no significant difference porosity among the groups ( *p* > 0.05).

Fig. 1 shows the total porosity of the scaffolds was 100%. This means that the scaffolds had a completed porous structure, allowing for the passage of fluids and nutrients. The average porosity of the scaffolds was higher than the porosity of the cancellous bone, which is a type of spongy bone tissue found in the human body. Cancellous bone typically has a porosity of around 79.3%. 16

**Fig. 2:**
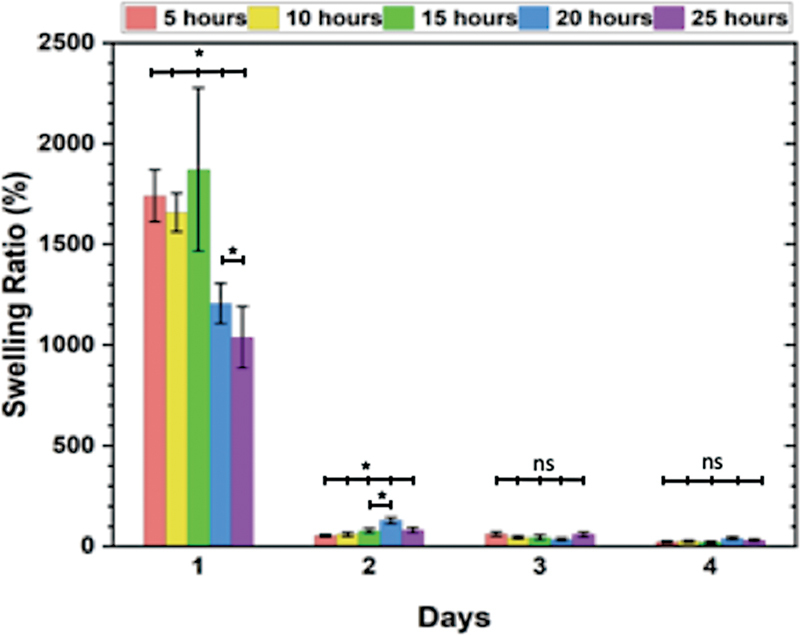
The swelling ratio of the hydroxyapatite (HAp)/bacterial nanocellulose (BN) three-dimensional (3D) scaffolds was evaluated at different time points for immersion technique. The results indicated a significant difference between the groups on day 1 and 2 ( *p* < 0.05).

The swelling ability of the scaffold is indeed crucial for hydrogel material, and Fig. 2 depicts the swelling ratio of the HAp/BN scaffolds over time. The results indicate that the swelling ratio increased on day 1 and then sharply decreased on day 2. There was a significant difference between the groups on both day 1 ( *p* < 0.05) and day 2 ( *p* < 0.05).

**Fig. 3:**
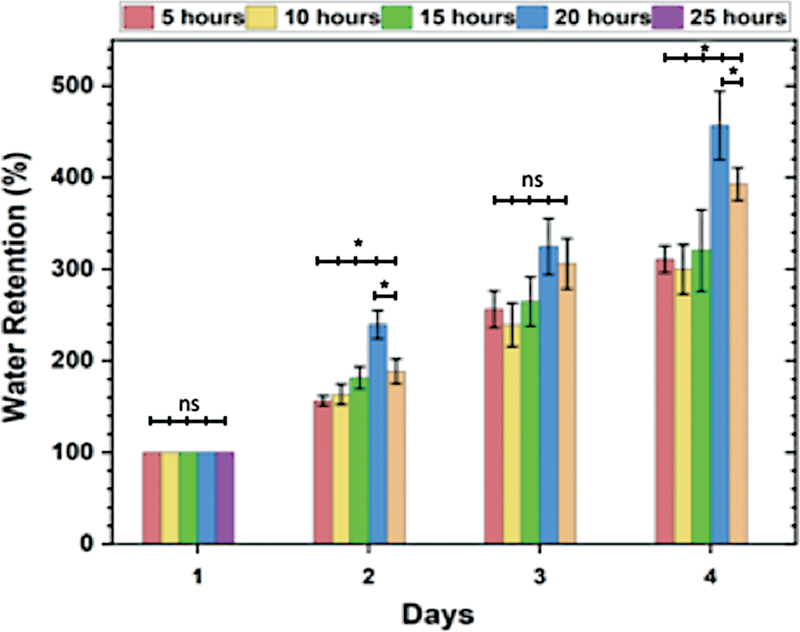
The water retention of hydroxyapatite (HAp)/bacterial nanocellulose (BN) three-dimensional (3D) scaffolds over time using different immersion times. The results indicate a significant difference between the groups on day 2 and day 4 ( *p* < 0.05).

The data presented in Fig. 3 shows the water retention capacity of the HAp/BN 3D scaffolds over a period of 4 days. The results indicate a gradual increase in water retention from day 1 to day 4 for all scaffolds groups. Notably, the scaffolds immersed in the HAp for more than 15 hours exhibited higher water retention compared to the other groups.

The statistical analysis showed a significant difference between the groups on day 2 and day 4 ( *p* < 0.05), indicating variations in water retention capacity based on the immersion time. The scaffolds with longer immersion times demonstrated improved water retention, which can be attribute to the enhanced bonding between the HAp and the scaffold matrix. This enhanced bonding delays the evaporation of absorbed water molecules, leading to increased water retention capacity. 17 18

**Fig. 4:**
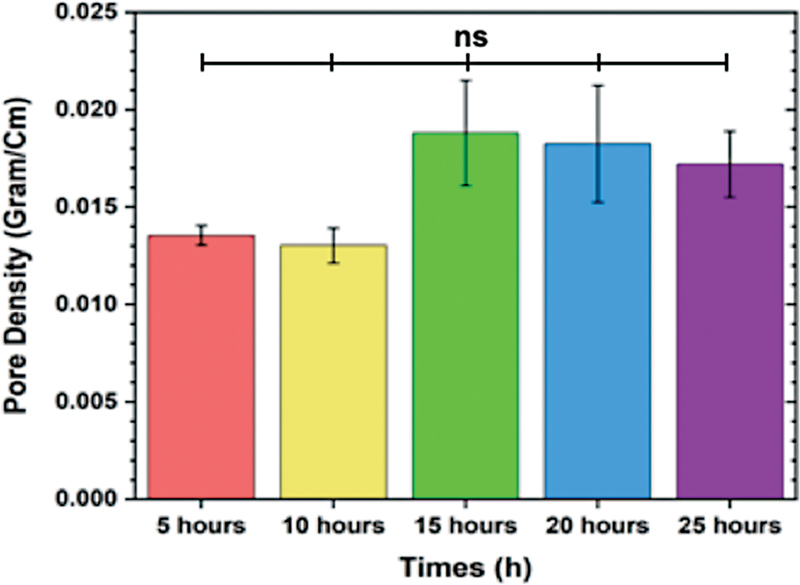
The pore density of hydroxyapatite (HAp)/bacterial nanocellulose (BN) three-dimensional (3D) scaffolds was evaluated for different immersion time using immersion technique. The data did not show a significant difference between the groups ( *p* > 0.05).

The scaffold immersed for more than 15 hours exhibited a higher pore density compared to those immersed for less than 15 hours ( Fig. 4 ). This higher pore density can contribute to faster water absorption by the scaffold. The data also suggests that the scaffold immersed for 15 hours had a higher water intake compared to the other groups, and it retained water for a longer duration.

**Fig. 5:**
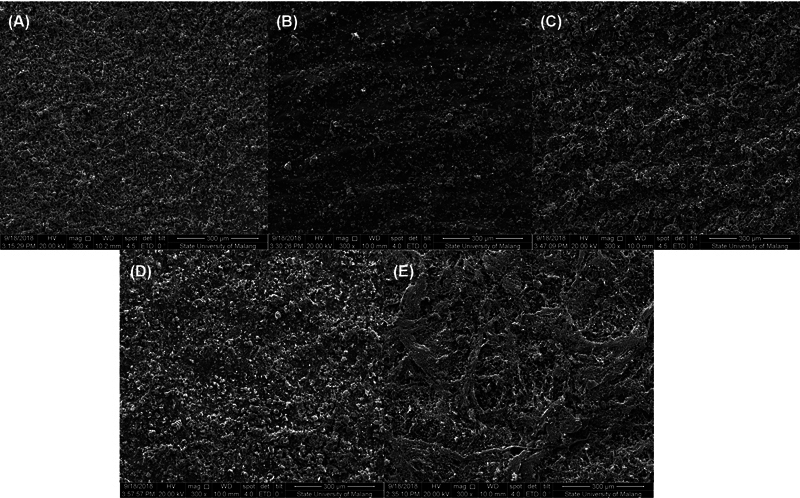
Scanning electron microscopy (SEM) morphology and pore distribution of scaffolds. ( **A** ) Five hours; ( **B** ) 10 hours; ( **C** ) 15 hours; ( **D** ) 20 hours; and ( **E** ) 25 hours.

Additionally, the data indicates that the 15-hour group had a higher count of pores in the diameter ranges of 101 to 350 μm (215), 351 to 500 μm (35), and > 501 μm (23). Conversely, the 5-hour group had a greater number of pores with a diameter of less than 100 μm (13,169) ( Fig. 5 ).

## Discussion

The porosity of HAp/BN scaffolds was also found to be similar to the porosity reported in the study by Gao et al. 17 In their study, the porosity range of other materials such as polylactic acid and poly (lactic-co-glycolic acid) was found to have porosity values ranging from 91 to 95%. 19 20 Porosity plays a critical role in bone regeneration as it enables the transport of nutrients, infiltration of tissue, and vascularization. These factors are crucial for supporting the growth and regeneration of bone tissue. The high porosity observed in the HAp/BN scaffolds suggests that they have the potential to facilitate these processes and promote bone regeneration. 3 21

The analysis of the swelling ratio in the study revealed some interesting observations. Initially, on day 1, there was an increase in the swelling ratio, which can be attributed to the scaffolds' ability to absorb liquid when soaked in PBS. However, on the second day, the swelling capacity of the scaffolds deteriorated. Despite this, the 20HD1 scaffold still exhibited a higher absorption rate of 128.8% than the other scaffolds. The findings are consistent with a previous study conducted by Rubina et al, 22 which suggested that the nanocellulose hydrogels exhibit nearly irreversible swelling after drying but can be repeatedly swollen. This indicates that the scaffolds used in the present study share similar characteristics with nanocellulose hydrogels in terms of their swelling behavior. 2 7

Interestingly, the finding of this study contradicts with the study by Gorgieva and Trček, 23 which stated that BN has low absorption ability due to the formation of strong intra- and interhydrogen bonds after drying. The HAp/BN scaffolds developed in this study showed improved swelling properties, indicating that the incorporation of NaOH and ammonium sulfate resulted in a more uniform porosity structure distribution and enhanced sensitivity toward ionic solutions. 22

The ability of the HAp/BN scaffolds to maintain their swelling capacity and exhibit a higher absorption rate suggests their potential for applications where water absorption and retention are essential, such as in tissue engineering and drug delivery systems. Further investigations on the scaffolds' swelling behavior and their response to different environmental conditions will provide a deeper understanding on their performance and suitability for specific applications. 23

The ability of the HAp/BN scaffolds to retain water is of great significance in various applications, particularly in tissue engineering and regenerative medicine. Maintaining an adequate hydration level is crucial for cell viability, nutrient transport, and overall tissue regeneration. The results suggest that optimizing the immersion time and incorporating HAp can enhance the water retention capacity of the scaffolds, providing a favorable environment for cellular activities and tissue regeneration processes. 23 24

The increased pore density in scaffolds immersed for longer durations may be attributed to the incorporation of HAp on the surface of the BN. 25 This incorporation of HAp can enhance the rigidity and tightness of the scaffolds' structure, leading to a slower absorption kinetics and reduced shrinkage. This phenomenon allows the scaffolds to retain water more effectively. 26 Furthermore, these findings suggest that the presence of HAp can improve the water absorption capacity and retention of the scaffold. The higher pore density observed in the scaffolds immersed for longer durations supports the hypothesis that the HAp content contributes to these properties. 27 28

Abbasi et al 29 found that the pore sizes between 200 and 350 μm were optimal for promoting osteoblast proliferation, while a larger pore size of 500 μm was effective in facilitating endothelial cell growth and promoting the secretion of proinflammatory cytokines such as tumor necrosis factor-α and interleukins 6, 10, 12, and 13. These cytokines have the potential to stimulate bone regeneration. 30

## Conclusion

In the field of tissue engineering, scaffolds play a crucial role and must trigger a slight immune response. To maximize nutrient transport and exchange, it also needs to be biocompatible, degrade at an acceptable pace, have sufficient porosity, and pore size with 100% interconnected pore. The scaffold's mechanical strength must be enough. Prefabricated porous scaffolds, decellularized extracellular matrices, cell sheets with secreted extracellular matrices, self-assembled hydrogels, and rapid prototyping are all methods that can be used to create scaffolds with improved reliability and functionality for dental tissue engineering. Our findings suggest that the HAp/BN 3D scaffold, especially when immersed in HAp for 15 hours, possesses promising physical properties that make it suitable for various applications in dental tissue engineering.
